# Crystal Structures of *E. coli* Native MenH and Two Active Site Mutants

**DOI:** 10.1371/journal.pone.0061325

**Published:** 2013-04-18

**Authors:** Jodie M. Johnston, Ming Jiang, Zhihong Guo, Edward N. Baker

**Affiliations:** 1 Maurice Wilkins Centre and School of Biological Sciences, University of Auckland, Auckland, New Zealand; 2 Department of Chemistry and State Key Laboratory for Molecular Neuroscience, The Hong Kong University of Science and Technology (HKUST), Kowloon, Hong Kong SAR, China; University of Canterbury, New Zealand

## Abstract

Recent revision of the biosynthetic pathway for menaquinone has led to the discovery of a previously unrecognized enzyme 2-succinyl-6-hydroxy-2,4-cyclohexadiene-1-carboxylate synthase, also known as MenH. This enzyme has an α/β hydrolase fold with a catalytic triad comprising Ser86, His232, and Asp210. Mutational studies identified a number of conserved residues of importance to activity, and modeling further implicated the side chains of Tyr85 and Trp147 in formation of a non-standard oxyanion hole. We have solved the structure of *E. coli* MenH (EcMenH) at 2.75 Å resolution, together with the structures of the active site mutant proteins Tyr85Phe and Arg124Ala, both at 2.5 Å resolution. EcMenH has the predicted α/β hydrolase fold with its core α/β domain capped by a helical lid. The active site, a long groove beneath the cap, contains a number of conserved basic residues and is found to bind exogeneous anions, modeled as sulfate and chloride, in all three crystal structures. Docking studies with the MenH substrate and a transition state model indicate that the bound anions mark the binding sites for anionic groups on the substrate. The docking studies, and careful consideration of the active site geometry, further suggest that the oxyanion hole is of a conventional nature, involving peptide NH groups, rather than the proposed site involving Tyr85 and Trp147. This is in accord with conclusions from the structure of *S. aureus* MenH. Comparisons with the latter do, however, indicate differences in the periphery of the active site that could be of relevance to selective inhibition of MenH enzymes.

## Introduction

Menaquinone (MQ) is an important component of the electron transport chain. In Gram-negative organisms such as *E. coli* that also contain ubiquinone (UQ), it functions in anaerobic respiration, whereas in Gram-positive bacteria where UQ is not present it functions in all forms of respiration [1], [2]. Recent work also suggests that demethylmenaquinone in *E. coli* functions during aerobic respiration [3]. Enzymes from the MQ biosynthesis pathway have been targets for drug design and MenA inhibitors have been developed that have been shown to inhibit the growth of the *M. tuberculosis* bacilli [4], [5]. The enzymes making up the standard “chorismate dependent” menaquinone biosynthesis pathway in *E. coli* have been identified over a number of years and currently comprise seven known members (MenA-MenF, MenH and UbiE) [1], [2], [6]. In recent years an alternative MQ biosynthesis pathway termed the “futalosine pathway” has been discovered in some organisms such as *Helicobacter pylori* and *Streptomyces coelicolor* that did not appear to have orthologues of the traditional MQ biosynthesis genes [1], [6], [7].

**Figure 1:**
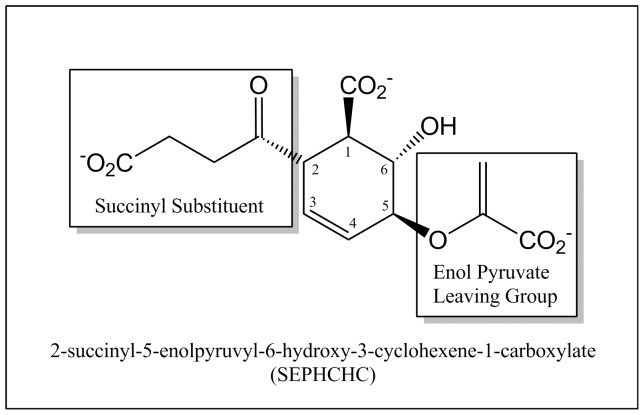
SEPHCHC substrate of the MenH reaction. The succinyl and enol pyruvate groups are highlighted in boxes. Numbering refers to the carbons on the ring.

**Figure 2:**
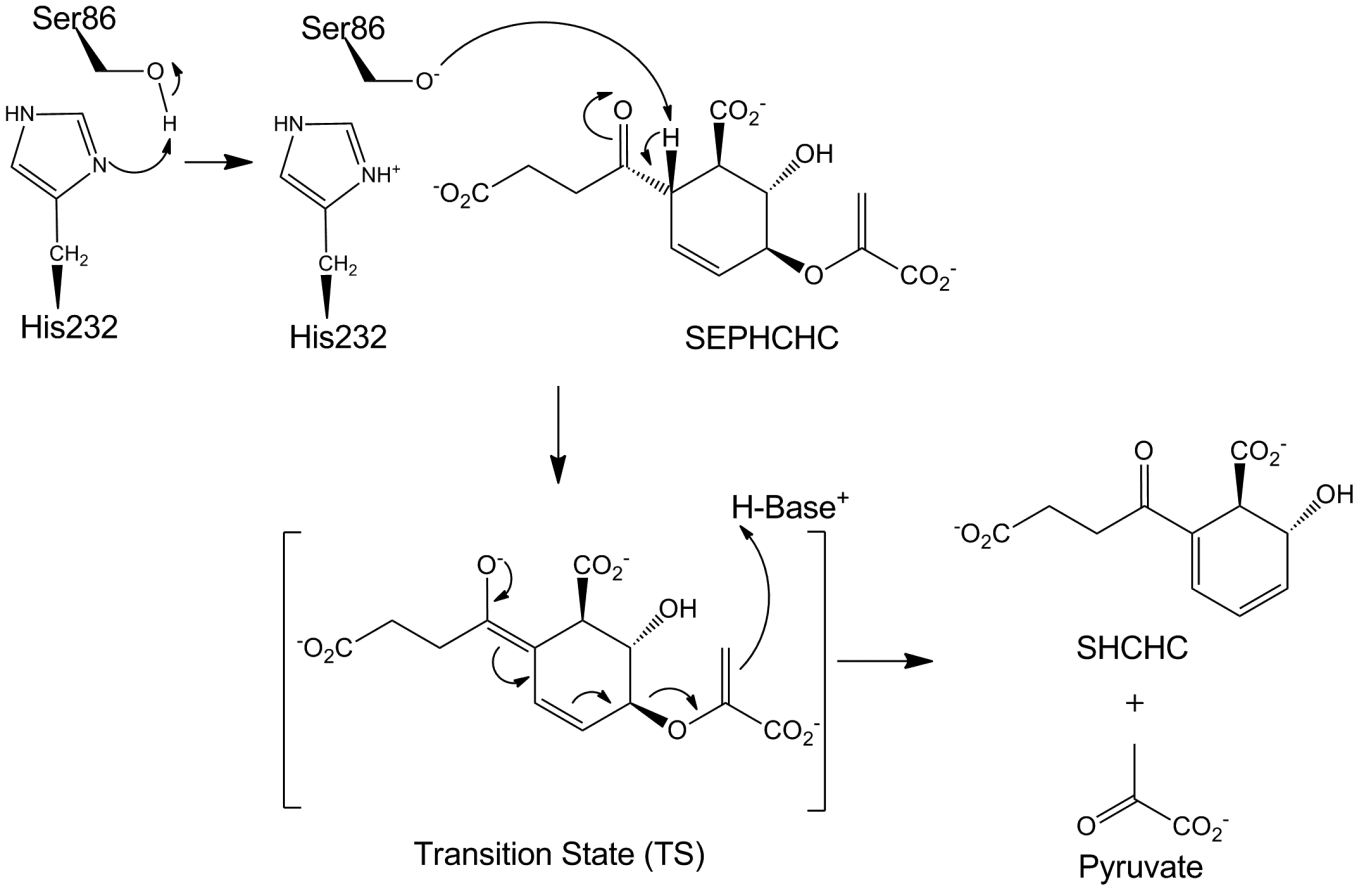
Current proposed mechanism of the MenH catalyzed reaction. Based on work from Jiang *et al.* [9] and Dawson *et al.* [12]). In this mechanism, Ser86 acts as a base, abstracting the proton from C2 of the ring after activation by the base His232, which abstracts the hydroxyl proton from Ser86. His232 may then also act as a proton donor for conversion of methylene group of the pyruvate substituent to methyl.

MenH was identified as a putative α/β hydrolase involved in menaquinone biosynthesis just over a decade ago but its actual role in the pathway has been revised several times. Its first annotation was as a naphthoyl-CoA thioesterase, acting subsequent to MenB and cleaving the CoA moiety from 1,4-dihydroxynaphthoate (DHNA) [2]. Re-annotation of the activity of an earlier enzyme in the pathway, MenD [8], then led to the recognition that the true enzymatic activity of MenH is to covert the MenD reaction product, 2-succinyl-5-enolpyruvyl-6-hydroxy-3-cyclohexene-1-carboxylate (SEPHCHC; Figure 1) to 2-succinyl-6-hydroxy-2,4-cyclohexadiene-1-carboxylate (SHCHC) by a 2,5 elimination of pyruvate from the cyclohexene ring [9], [10]. MenH is thus correctly annotated as SHCHC synthase.

Analysis of 47 MenH sequences across a range of species revealed that only 15 residues are absolutely conserved between them [9]. Mutational analyses then led to proposed roles for most of these residues in either substrate binding or catalysis, including three residues that form the catalytic triad typical of α/β hydrolases (Ser86, His232 and Asp210), four basic residues (Arg90, Arg124, Arg168 and Lys212), two aromatic residues (Trp147 and Tyr85) and one acidic residue (Asp128) [9]. In the current MenH mechanism, Ser86 is activated by His232 by removal of its hydroxyl proton, and then acts as a base, abstracting a proton from C2 of the SEPHCHC ring **(** Figure 2 **)** [9]. The reaction intermediate carries a negative charge that is stabilized by the enzyme. In the case of SEPHCHC this negative charge should be concentrated on or around the carbonyl oxygen of the 2-succinyl group. In general, α/β hydrolases stabilize their catalytic intermediates by formation of an oxyanion hole using two backbone amides – one from the residue subsequent to the catalytic serine and another variably positioned but often located on a loop linking a β-strand to the first helix [11]. When *E. coli* MenH (EcMenH) was re-annotated it was postulated that, instead of the traditional oxyanion hole typical of α/β hydrolases, MenH utilizes an “unconventional oxyanion hole” formed by the side chains of the conserved Trp147 and Tyr85 [9]. A recent analysis of the structure of *Staphylococcus aureus* MenH (SaMenH) suggests, however, that at least in this case a traditional oxyanion hole is used [12].

Two structures are currently available for presumed MenH enzymes, from *S. aureus* [12] (1.94 Å resolution; PDB code 2×mz) and from *Vibrio cholerae* (1.9 Å resolution, PDB code 1r3d, J. Gorman & L. Shapiro, unpublished). No biochemical studies have been reported on these enzymes, however, and the *E. coli* enzyme remains the only MenH to have been biochemically characterized. Accordingly, we report the structure of the MenH from *E. coli* at 2.75 Å resolution, along with the structures of two active site mutant enzymes, Arg124Ala and Tyr85Phe, both at 2.5 Å resolution. We show that bound anions in the active site identify key anchor points for the anionic SEPHCHC substrate. Docking studies and the location of a conserved water molecule further point to the likelihood that EcMenH utilizes a conventional oxyanion hole rather than the proposed non-conventional one. Comparison of the EcMenH structure with the two previously reported structures also reveals an intriguing pattern of substitutions that could be exploited in selective inhibition of MenH enzymes.

## Methods

### Cloning, Expression and Purification

Plasmids containing hexa-histidine tagged native and mutant (Tyr85Phe and Arg124Ala) EcMenH constructs, made as previously described [9], [10], were transformed into BL21 (DE3) cells. These constructs have an additional 16 residues, MHHHHHHSSGLVPRGS at the N-terminus, providing a hexahistidine affinity tag. The resulting transformants were plated onto an LB–agar plate containing ampicillin at 100 mg ml^−1^, and cultures were grown overnight in MDG media [13], then used to inoculate pre-sterilized ZYM-5052 media containing ampicillin at 100 mg ml^−1^. Cells were grown at 310 K for 3 h then at 298 K overnight for protein overexpression using autoinduction [13].

Bacterial cell pellets containing overexpressed His-tagged EcMenH (native and mutants) were resuspended in 20–30 ml lysis buffer (50 mM Tris–HCl pH 8.5, 150 mM NaCl, 20 mM imidazole, 1% v/v glycerol, 1 mM TCEP) with a complete EDTA-free protease-inhibitor tablet, lysozyme, DNase and RNase added, and then lysed by cell disruption (Constant Cell Disruption Systems), followed by pelleting of the insoluble material by centrifugation (20 000 g for 20 min). The supernatant containing soluble His-tagged EcMenH was then loaded on to a 5 ml Ni^2+^ HiTrap affinity column pre-equilibrated in lysis buffer, washed with 6–10 column volumes of lysis buffer and eluted with a gradient to 100% elution buffer (50 mM Tris–HCl pH 8.2, 150 mM NaCl, 500 mM imidazole, 1% v/v glycerol, 1 mM TCEP). His-tagged EcMenH (native and mutants) was then further purified by size-exclusion chromatography using a Superdex 200 16/60 column in 50 mM Tris–HCl pH 8.5, 150 mM NaCl, 1% v/v glycerol, 1 mM TCEP. The protein eluted at a size consistent with a monomer. Protein samples were also analyzed by dynamic light scattering (DynaPro, Protein Solutions) and SDS–PAGE.

### Crystallization

Initial crystallization conditions were found by sitting-drop vapor diffusion (100 nl protein solution +100 nl precipitant) using a Cartesian Honeybee dispensing robot with an in-house 480-component screen [14]. Reproducible diamond shaped crystals, referred to here as Type 1, were grown from subsequent optimizations of the initial trisodium citrate, ammonium/lithium sulfate condition, by hanging-drop vapor diffusion in 24-well plates with siliconised coverslips (Hampton Research), using a range of drop sizes from 1 µl upwards. Crystals were grown from a protein solution comprising 20–26 mg ml^−1^ EcMenH in 50 mM Tris–HCl pH 8.5, 150 mM NaCl, 1% v/v glycerol and 1 mM TCEP, mixed with precipitants containing 0.15 M Tri sodium citrate pH 5.2–5.9 and varying amounts of ammonium sulfate (0.05–1.25 M) and lithium sulfate (0–0.75 M). Subsequently, when the optimization crystal trays were rechecked 10 months later, crystals of a different rod-shaped morphology (Type 2 crystals) were found alongside the Type 1 crystals in several native and Tyr85Phe mutant crystallization drops. These most likely resulted from loss of water over time; the drop volume was noticeably reduced and there was no evidence of proteolysis in the subsequent structure determination.

### Data Collection and Processing

For data collection, crystals were soaked in cryoprotectant and flash-frozen in liquid N_2_. Cryoprotectant screening suggested that mother liquor with increased ammonium sulfate (to 1.0 M) and lithium sulfate (to 1.5 M), supplemented with a small amount of ethylene glycol (2%), gave the best protection. Data were collected at the Australian Synchrotron on either beamline MX1 (with an ADSC Quantum 315R CCD detector) or MX2 (with an ADSC Quantum 210r CCD detector). Complete data sets were collected for the native Type 1 crystals (MX2, λ = 0.9592 Å), Arg124Ala Type 1 crystals (MX1 λ = 0.9537 Å) and Type 2 Tyr85Phe crystals (MX1, λ = 0.9537 Å). The Type 1 crystals proved to belong to space group I4_1_22, with 3 molecules per asymmetric unit, whereas the Type 2 crystals had space group C2, again with 3 molecules per asymmetric unit (**Table 1**).

**Table 1:** Data Collection and Processing Details.

Structure	MenH_Native	MenH_R124A	MenH_Y85F_C2
Wavelength (Å)	0.9592	0.9537	0.9537
Crystal-detector dist. (Å)	450.0	249.9	200.1
Mosaicity (deg)	0.305–0.665	0.138	0.136
Δphi (no. of frames)	0.5 (990)	0.5 (360)	1.0 (366)
Exposure time (s)	1.0	2.0	1.0
Space Group	I4_1_22	I4_1_22	C2
Cell Parameters	*a = b = *116.7,*c = *476.7 Å*;* *α = β = γ = *90.0 deg	*a = b = *115.1,*c = *469.8 Å*;* *α = β = γ = *90.0 deg	a = 250.5, b = 41.6,c = 93.1 Å,*α = γ = *90.00, *β = *95.9 deg
*% solvent*; Matthews coefficient; no. mols/AU	*73.6*; 4.66; 3	*72.5*; 4.47; 3	55.7; 2.77; 3
Resolution range (Å)^A^	100–2.75(2.85–2.75)	92.76–2.50(2.64–2.50)	124.59–2.49(2.62–2.49)
R*merge* (%)^A^	15.4 (93.4)	19.0 (184.7)	32.5 (95.7)
Rpim (%)^A^	–	5.1 (48.7)	12.9 (37.6)
No. unique reflections	43479	54795	34116
Mean I/σI A	22.6 (3.5)	17.6 (1.8)	5.9 (2.0)
Multiplicity	17.5 (10.4)	14.9 (15.2)	7.3 (7.3)
Completeness (%)^A^	99.9 (100)	99.6 (99.2)	99.7 (98)
Program	HKL2000	XDS/SCALA	XDS/SCALA

A Values in parentheses are for outer resolution shell.

For the native EcMenH crystals, data were processed using DENZO and scaled with SCALEPACK in HKL-2000 [15]. A highly-redundant data set, free of radiation damage, was obtained by combining three data sets obtained from different parts of the same crystal. For the mutant crystals the raw data were processed with XDS [16], [17] and space group possibilities analyzed using POINTLESS [18]. Data were scaled using SCALA in the CCP4 suite [18], [19]. Full details of the best data sets are given in Table 1.

### Structure Solution and Refinement

For native EcMenH, a partial molecular replacement solution was found in Phaser in the *CCP*4 suite [19], [20] using a Balbes [21] modified version of the *V. cholerae* MenH (VcMenH) structure (PDB code 1r3d, J. Gorman & L. Shapiro, unpublished) as a search model. Three molecules were found in the asymmetric unit corresponding to a high solvent content of ∼73% (Table 1). Two of these molecules were positioned correctly with well-defined density. After improving the model for one of the correctly positioned molecules by manual building in Coot [22] another round of molecular replacement was undertaken in Phaser using this improved molecule as the search model. The resulting three molecules were now positioned correctly and were refined through subsequent rounds of manual model building in Coot and refinement in Refmac5 [23] and Buster 2.10.0 [24]. Solvent molecules were added automatically but were only retained if they had appropriate shape, hydrogen bonding interactions, and B-factors. Two such solvent molecules were modeled as ions, one sulfate and one chloride, after such examination. Non-crystallographic symmetry restraints were applied to the 3 protein molecules, but translation-libration-screw (TLS) parameterization was not used. Structures for the Arg124Ala and Tyr85Phe mutants were solved by molecular replacement with Molrep [25] using the native EcMenH structure as a search model. The Tyr85Phe structure, solved in space group C2 (Type 2 crystals) also had 3 molecules in the asymmetric unit, but with a lower solvent content of 56%. The mutant structures were refined in a similar manner to that described above, Final refinement and model statistics for all structures are given in Table 2. Model quality was checked with Molprobity [26] and figures were prepared using Pymol [27], [28].

**Table 2:** Refinement Statistics.

Structure	MenH_Native	MenH_R124A	MenH_Y85F
PDB Code	4GDM	4GEC	4GEG
Resolution Range (Å)	45.77–2.75	81.42–2.50	33.71–2.49
No of reflections	43432	54795	34093
R (%) (No of reflections)	19.3 (41250)	19.4 (52003)	17.7 (32370)
R*_free_* (%) (No of reflections)	22.8 (2182)	22.3 (2792)	24.9 (1723)
**R.m.s.d from ideal**			
Bond lengths (Å)	1.07	1.06	1.07
Bond angles (°)	0.01	0.01	0.01
Ramachandran (most favored; outliers) A	(97.25; 0.00)	(98.56; 0.00)	(97.91; 0.00)
Molprobity score/percentile^A^	2.10/97^th^	2.04/95^th^	1.83/98^th^
Wilson plot B-factor (Å^2^)	69.9	61.5	34.7
Cruickshank’s DPI (Å)^B^	0.29	0.22	0.39
**Model details**			
Protein	5949	5923	5959
Waters	65	232	419
Small molecule/ions	3 chloride ions; 1 glycerol;4 sulfate ions	3 chloride ions; 1 glycerol; 1 ethylene glycol; 4 sulfate ions	7 chloride ions; 1 glycerol; 1 ethylene glycol; 13 sulfate ions
**Mean ** ***B*** ** factors** (Å^2^)			
Protein	53.7	49.1	20.9
Waters	42.4	45.6	21.4
Sulfate (active site sulfates)	79.8 (72.6)	71.1 (71.4)	64.1 (34.6)
Chloride	53.0	46.1	29.5

A Ramachandran regions and scoring as defined in Molprobity [26].

B DPI Cruickshank’s DPI for coordinate error based on *R* factor (Å) from Buster 2.10.0 [24].

### Substrate Docking

**Figure 3:**
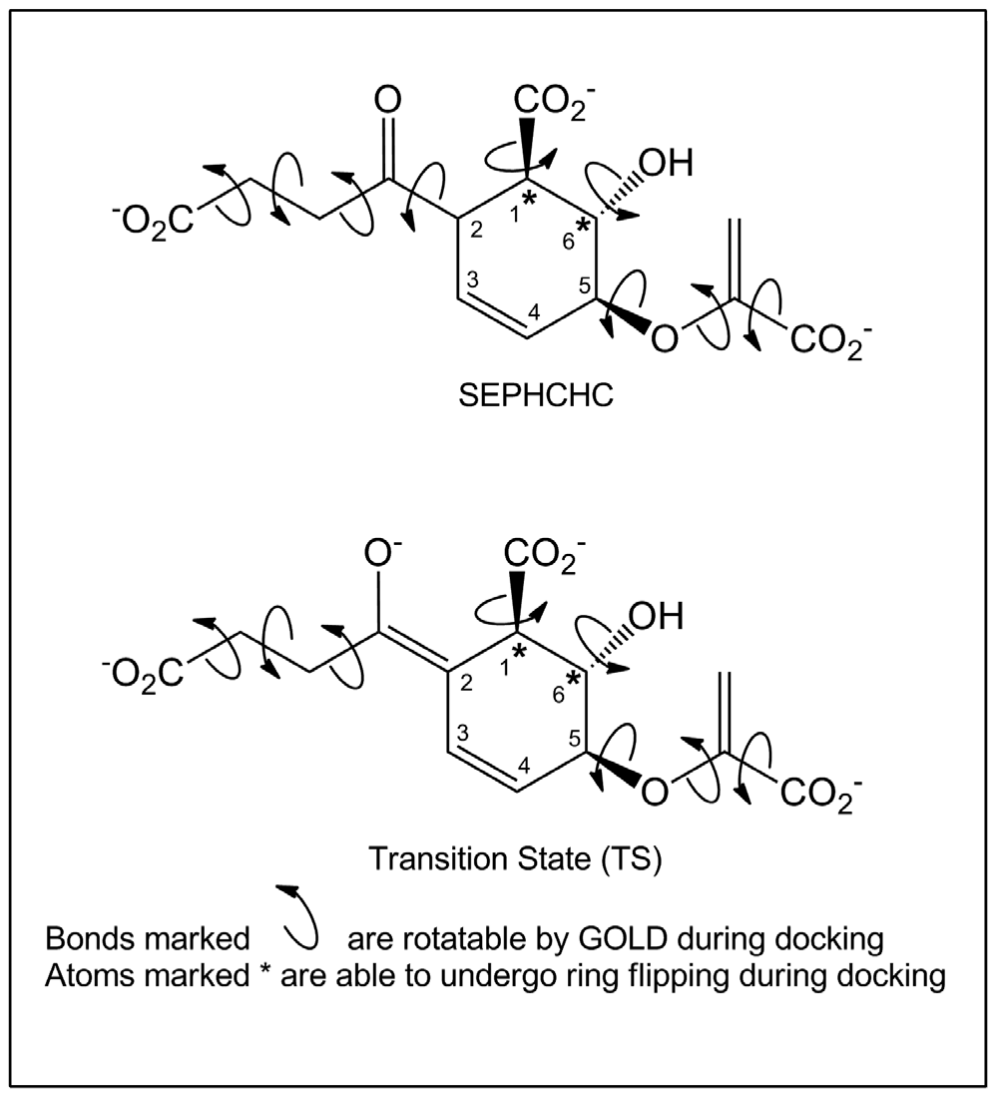
Structures used for docking to MenH. Chemical structures of the substrate (SEPHCHC) and transition state (TS) models used in docking studies to MenH. Bonds marked with a curly arrow were defined as rotatable in GOLD, and the atoms marked with an asterisk were able to undergo ring flipping.

The structures of the substrate SEPHCHC and a transition state model (TS) (Figure 3), with hydrogen atoms included, were generated for docking with ChemBio3D v12.0 (Cambridgesoft) using the MMFF94 force field for energy minimization. The carboxylate groups and the enolate oxygen on these structures were deprotonated, as appropriate for pH 8.5, and this charge allocation was further defined by the docking program to allow delocalization of the negative charge across these bonds. Docking experiments were performed on the native, Arg124Ala and Tyr85Phe mutant crystal structures of MenH in GOLD v5.1 [29] using a standard genetic algorithm (GA) protocol to explore the conformation/orientation space. Docking used the default GOLD settings unless otherwise specified below. The active site cavity was defined as a volume that encompassed all the conserved active site residues and the sites of the sulfate and chloride anions, which were removed from the model. Ligand flexibility was permitted in the docking experiments by allowing rotation about single bonds of the ring substituents, as indicated in Figure 3, and changes in the ring pucker. Independent docking trials were carried out for both the TS and SEPHCHC, conducted separately for each of the three chains present in each crystal structure. The following experiments were carried out for each of the 9 chains: docking experiments with no constraints, leaving all protein side chains as observed in the crystal structure; experiments with no constraints allowing flexibility of key active site residues Ser86, Arg90, Arg124 and Arg168 [30], and the same two experiments carried out but incorporating a distance constraint of 3 Å between the Ser86 OG and the C2 of the SEPHCHC. For each of the docking trials, solutions were ranked according to the value of the GOLD-Score fitness function.

## Results

### Overview of the Crystal Structures

The final model refinement statistics for the best native and mutant structures are given in Table 2. Each of the structures contains three independent molecules (A, B and C) in the asymmetric unit but EcMenH appears to be monomeric in solution on the basis of size exclusion chromatography and dynamic light scattering (results not shown). Analysis of the structures with PISA [31] supports this conclusion; none of the interfaces between molecules in the crystal have a score significant enough to suggest any biological relevance. Structural comparisons with SSM [32], between the three molecules of each structure as well as between the native and mutant structures show that they are all very similar, with root-mean-square (rms) differences of 0.34–0.61 Å in Cα-atom positions over the entire protein length. In each case unbroken electron density is seen for at least residues 1–251 of the 252-residue protein. In addition, 5–6 residues of the N-terminal His_6_ tag could be modeled for two of the chains in all the I4_1_22 structures and in all chains of the Tyr85Phe C2 mutant structure. Each structure also contains a number of small molecules; sulfate ions, chloride ions, ethylene glycol and glycerol. Most are distributed over adventitious sites on the protein surface and have no apparent functional significant, but one sulfate ion and one chloride ion are consistently present in the active site, as described later.

### The EcMenH Fold

**Figure 4:**
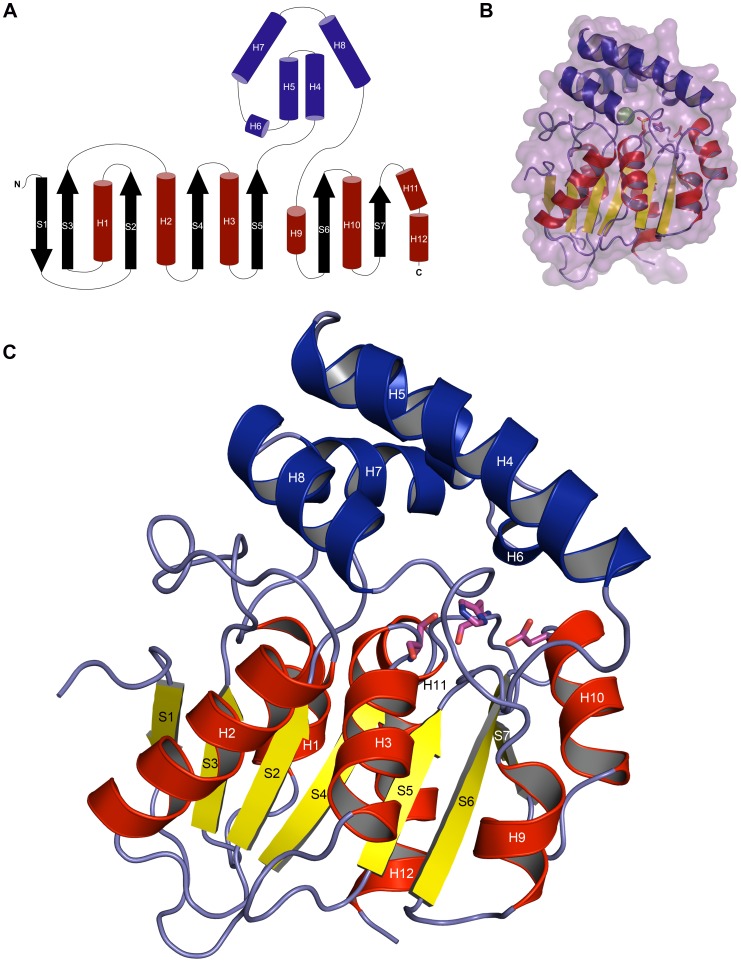
The EcMenH structure. (A) Topology diagram (B) Semi-transparent surface representation overlaying a cartoon representation as shown in c. Sulfate and chloride ions in the active site are depicted as sticks and a green sphere respectively (C) Cartoon representation. Helices and β-strands are labeled H and S respectively, secondary structure elements of the core α/β domain are colored red (helices) and yellow (β-strands) and those of the helical lid domain are in blue. The catalytic triad is depicted in stick representation in magenta.

EcMenH adopts a typical α/β-hydrolase fold, comprising a core β-sheet of mostly parallel strands surrounded on both sides by **α**-helices (Figure 4) [33], [34], [35]. In a similar manner to that seen in *M. tuberculosis* Rv0554 [36], SaMenH [12] and VcMenH (PDB code 1r3d), EcMenH has seven β-strands compared with the usual eight in other α/β-hydrolases and a helical cap or “lid” domain made up of five helices (H4–H8; with H6 being a short 3^10^ helix in EcMenH) that are contributed from the middle third of the protein (residues ∼115–188) (Figure 4). The presence of a helical lid domain is a common adornment to the α/β-hydrolase fold although its components are variable [34], [35]. In the case of EcMenH the helical lid sits over the loops containing the active site catalytic triad residues and form part of the active site cavity.

### Active Site

#### The catalytic triad

As is characteristic of the α/β-hydrolases the three catalytic triad residues (Ser86, His232, Asp210) are located on loops of the core α/β–domain (Figure 4C) [33]. The catalytic serine is located within a GXSXG motif that forms a tight turn between S4 and H3, known as the ‘nucleophile elbow’ in which the serine adopts unusual Ramachandran φ/Ψ angles. In the crystal structures, the serine side chain shows some flexibility, adopting several alternative rotamers in the various chains of the three crystal structures. The distance between the serine hydroxyl and His232 NE1 varies between chains from 2.5–3.2 Å, reflecting this flexibility. His232 is located on the long S7–H11/H12 loop, with its imidazole side chain locked in place by extensive hydrogen bonding interactions, including hydrogen bonds between its ND1 and the OD1 of the third residue of the catalytic triad, Asp210. The ring CH groups of His232 are also involved in two geometrically favourable C-H…O hydrogen bonds; one between CD2 and the OH of Tyr85 (3.1–3.4 Å in different chains), another between CE1 and the main chain carbonyl oxygen of Gly110 (2.9–3.4 Å in different chains). Dawson *et al*. postulate that the Tyr85-His232 interaction, which is seen in SaMenH just as it is here, may help to orientate the positive charge on the histidine when protonated and hence support its catalytic function [12]. Asp210 is located in the S6–H10 loop, on a tight turn with its position stabilized by backbone interactions between its carbonyl O and NH groups and backbone O or NH groups from residues 212–214 and 207. The side chain of Asp210 is shielded from the active site by the hydrophobic side chains of Phe213 and Val152.

**Figure 5:**
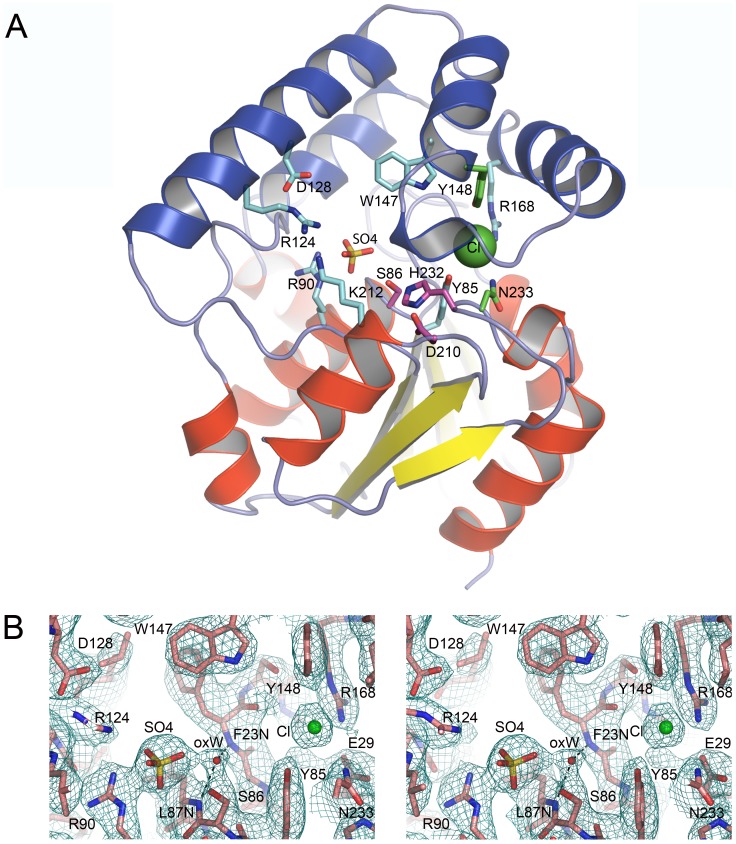
Features of the active site cavity. (A) Overview of the entire active site region, shown for chain A of the native protein. EcMenH is depicted in cartoon representation with key residues in stick form labeled with the one-letter amino acid code. Residues of the catalytic triad are in magenta, other fully conserved residues in cyan and additional residues that interact with bound ions are in green. The sulfate ion is in stick mode in orange and the chloride ion is shown as a green sphere. (B) Electron density for the sulfate and chloride ions in the active site, shown in a bias-removed 2Fo-Fc omit map, calculated after removal of the sulfate and chloride ions from the model. The water molecule occupying the classical α/β-hydrolase oxyanion hole, oxW, is also clearly visible, hydrogen bonded (broken lines) to 23 NH and 87 NH.

The structure also contains candidates, the main chain NH groups from Leu87 and Phe23, for the classical oxyanion hole of the α/β hydrolases [34]. In most chains of the native and mutant EcMenH structures there is a conserved water molecule located within a suitable distance and orientation to form hydrogen bonds with the NH groups forming this “traditional oxyanion hole” (Figure 5B). This water, which has a relatively low B-factor comparable with the surrounding protein structure, is also close (2.4–2.8 Å) to the conserved sulfate ion seen in the active site and discussed later.

#### Active site cavity

The active site cavity is formed between residues from the helical lid domain and loops and helices from the α/β core domain that face the lid domain (Figure 5A). The cavity formed between these two regions is an elongated groove that extends from one side of the core domain to the other with the catalytic triad in the middle. Analysis of this cavity using CASTp [37] reveals it to be on average ∼1200 Å^3^ in volume (994 to 1338 Å^3^ depending on the particular chain analyzed).

Sequence comparisons identified ten non-glycine residues that are completely invariant in 47 available MenH sequences [9]. These ten residues comprise the three members of the catalytic triad, Ser86, His232 and Asp210, together with seven other residues. These residues, Tyr85, Arg90, Arg124, Asp128, Trp147, Arg168 and Lys212, are all located within the active site cavity where they are presumed to play key functional or structural roles.

At the wider end of the active site cavity, the likely entrance, Arg124 from α**-**helix H4 of the lid domain faces Lys212 and Arg90 from α**-**helices H10 and H3 of the α/β-domain. Lys212 and Arg124 project into the cavity in a way that suggests that their primary role is to interact with the substrate. Lys212 does not contact any other protein group. Arg124 is held in place by a hydrogen bond between NE and the carboxyl group of Asp128, but has its NH1 and NH2 groups pointing out into the middle of the cavity. The side chain of Arg90, in contrast, tightly held to one wall of the cavity through hydrogen bonds with the main chain carbonyl oxygen of Thr183 and side chain amide OE1 of Gln188.

Further into the cavity are the residues of the catalytic triad, together with Tyr85, all of which are contributed by the catalytic α/β-domain. The Tyr85 aromatic ring is packed between the side chains of Ser86 and His235, while its OH group is hydrogen bonded to a water molecule and the catalytic His232 (a C-H…O bond), and interacts with the bound chloride ion (3.1–3.3 Å). Across the active site cavity, the side chain of Trp147, from helix H4 of the lid domain, is at the centre of a large cluster of aromatic residues comprising Phe23, Trp131, Phe144, Tyr148, Phe153 and Phe185. Its indole NH group is oriented into the solvent region of the cavity, towards Tyr85, but its distance from the Tyr85 OH (∼ 7.5 Å) seems too great for these two groups to form the proposed non-conventional oxyanion hole.

At the inner end of the cavity is Arg168, which is part of a network of residues that show an intriguing pattern of variation in different species, described later. The Arg168 side chain is well defined and held in place by multiple interactions: its NH2 atom interacts with the chloride ion (3.1–3.6 Å) and forms a hydrogen bonded ion pair with OE2 of Glu29; NE is hydrogen bonded to the OH group of Tyr147; and NH1 makes several hydrogen bonds with well-ordered water molecules.

#### Anion binding sites

A notable feature of the active site cavity is the presence of two anions that are consistently found bound in all EcMenH structures, occupying the same sites in each (Figure 5A). These two ions were modeled as sulfate and chloride, respectively, based on the size and shape of their electron density (Figure 5B), and high concentrations of sulfate (>1.0 M) in the crystallisation medium and 150 mM sodium chloride in the protein buffer. Further validation of their identity came when they were modeled as water molecules; at both sites the B factors assumed very low values, 2–3 fold lower than the surrounding protein side chains, and there was residual density in Fo-Fc maps that could not be accounted for by water molecules. The nature of the sites, both of which included neighbouring positively charged groups, also favoured their interpretation as anions, and when modeled as sulfate and chloride they were found to make much better hydrogen bonding contacts with the surrounding structure.

**Figure 6:**
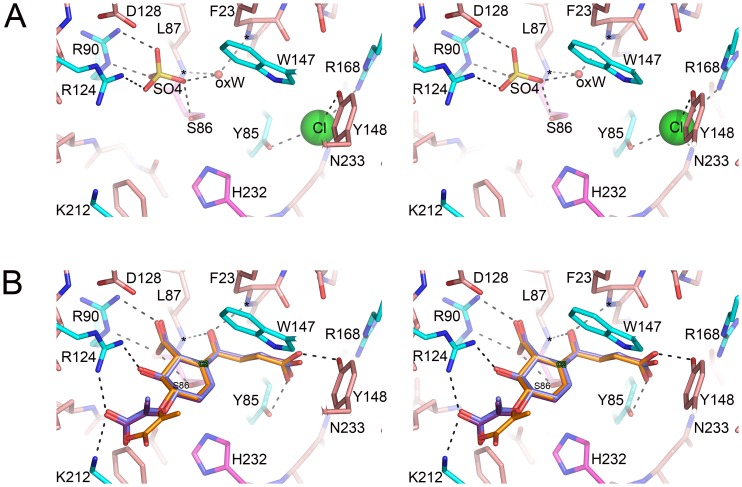
Ligand binding in the MenH active site. (A) Solvent species bound in the active site of native EcMenH. Residues labeled with the one-letter code. A water molecule (oxW) occupies the oxyanion hole typical of α/β-hydrolases, hydrogen bonded to the main chain NH groups of residues 23 and 87. A sulfate ion (yellow, stick representation) is bound to R90, R124, S86 and oxW, and a chloride ion (green sphere) is bound to Y85, Y148, R168 and N233. (B) Superposition of the three top solutions for the docking of the TS model into chain A of native EcMenH. The TS model is shown in stick representation, with the C2 carbon labeled. The active site base, Ser86, is almost directly below C2. Residues of the catalytic triad are in magenta, other fully conserved resides in cyan, and hydrogen bonds shown with broken lines.

Given that the SEPHCHC substrate (Figure 1) has three anionic groups, the carboxyl groups of the enol-pyruvate moiety, the SEPHCHC ring, and the succinyl moiety, it seems likely that the bound anions in the EcMenH crystal structure indicate key substrate interaction sites. The sulfate ion forms hydrogen bonded ion pairs with both Arg90 and Arg124, which appear to be the primary determinants of binding (Figures 5B, 6A). Arg90 makes a bidentate interaction through both NE and NH2, and appears to be a particularly important active site component, having a highly conserved orientation in all EcMenH structures. The sulfate ion makes additional hydrogen bonds with active site water molecules, including the water molecule located in the traditional oxyanion hole site, and (in most structures) with the side chain hydroxyl group of the catalytic Ser86.

The chloride ion is located further into the active site pocket, past the catalytic triad (Figures 5B, 6A). The chloride ion makes one charge interaction, with NH2 of Arg168, and additional interactions at distances of 2.9–3.4 Å from the OH groups of Tyr85 and Tyr148 and the side chain ND2 of Asn233. The consistent occupation of this site by an anion again suggests it is likely to be utilized by one of the carboxylate groups of the SEPHCHC substrate.

### Substrate Docking

*In silico* docking experiments were undertaken in which the substrate SEPHCHC and a transition state model TS (Figure 3) were docked to both native and mutant structures using GOLD. The SEPCHC docking experiments, in which rotation about single bonds in the substrate, and in the side chains of Ser86, Arg90, Arg124 and Arg168, was allowed, identified two possible binding modes. In one of these the succinyl group was oriented into the chloride binding site, and in the other the substrate was inverted, with its pyruvate group in the chloride site. Only the “succinyl in” binding mode, however, was consistent with the currently-accepted mechanism (Figure 2), with its C2 atom positioned to allow proton abstraction by the catalytic Ser86 hydroxyl [9], [12]. Imposition of a distance constraint of 3.0 Å between these two atoms, however, resulted in >90% of the solutions belonging to the “succinyl-in” pose.

Docking experiments with the TS model, in which the configuration about C2 was planar, gave much more consistent solutions. Even without constraints, almost all solutions adopted the “succinyl in” mode and positioned the negatively-charged enolate oxygen within hydrogen bond distance of the main chain NH groups of Phe23 and Leu87, which correspond to the classical oxyanion hole of the α/β hydrolases [34]; see Figure 6a. For the native structure, for example, TS docking with no distance constraints resulted in all solutions being “succinyl-in” for chains A and C, together with the top 3 solutions for chain B. In each case the top solutions agreed to within an rms deviation of 1.5 Å. All solutions also positioned the succinyl enolate oxygen into (or very near) the traditional oxyanion hole site.

Close inspection of the TS docking solutions shows consistent features in the interactions between the TS intermediate and conserved active site residues (Figure 6b). The succinyl carboxyl group sits between Tyr85 and Tyr148, in the site occupied by the chloride ion in the crystal structure (Figure 6A), hydrogen bonded to the phenolic oxygens of both residues. It is also within weak hydrogen bonding distance of the Trp147 indole NH and close enough to Arg168 (3.6–4.5 Å away on average) for some electrostatic interaction. Only when free rotation of the Arg168 side chain is allowed can it come close enough to the succinyl carboxyl group for direct hydrogen bonding, however. The negatively-charged enolate oxygen in this TS intermediate occupies the “traditional oxyanion hole” site, accepting hydrogen bonds from the main chain NH groups of Phe23 and Leu87 (Figure 6B). The SEPHCHC ring carboxyl group is consistently hydrogen bonded to Arg90 NE and NH2, in a bidentate interaction that mirrors the interaction of the sulfate ion with this residue. This supports the conclusion from mutational studies [9] that the conserved Arg90, which is tightly held to the wall of the active site, is crucial to substrate binding. The ring hydroxyl group is in most cases hydrogen bonded to the side chain of Arg124, and the hydrophobic part of the ring docks near the hydrophobic residues Leu153, Val152 and Trp147. There is more variability in the positioning of the pyruvate substituent in the outer pocket but its carboxyl group is in most poses hydrogen bonded to Arg124 and/or Lys212, both of them conserved residues.

### R124A and Y85F Mutants

Structural comparisons of native EcMenH with the two mutant structures, R124A and Y85F using SSM [31] shows they overlay very closely, with rms differences of 0.26–0.61 Å in Cα-atom positions over the entire structure. This shows that the mutations do not affect the polypeptide conformation significantly. The active sites differ very little, apart from weaker density for the sulfate ion in the R124A structure, due to the loss of the Arg124 interactions. The substrate docking experiments show few differences between native and mutant structures. The main difference for the R124A mutant is that much more variability is seen in the orientation of the pyruvate group, with the loss of the guanidinium group of Arg124. For the Y85F mutant, the contact between the succinyl carboxyl group and the phenolic oxygen of Tyr85 is necessarily lost, with the carboxyl group then being positioned closer to Tyr148 OH and Trp147 NE than in the other structures; it is consistently hydrogen bonded to both.

### Structural Comparisons

Searches of the Protein Data Bank using SSM [32] showed that by far the closest structural homologues of EcMenH are the two other presumed MenH enzymes, from *V. cholerae* (VcMenH; PDB code 1r3d, unpublished) and *S. aureus* (SaMenH; PDB code 2xmz, [12]). EcMenH and VcMenH share ∼41% sequence identity and have an rms difference of 1.26 Å over 238 common Cα-atom positions, the only significant conformational differences being in loops at the bottom of the core α/β domain, remote from the lid domain and the active site. EcMenH and SaMenH have lower sequence identity (29%) and poorer agreement in their polypeptide conformations, with an rms difference of 1.51 Å over 226 common Cα-atom positions. Nevertheless, the sequence and structural similarity supports the view that these three structures all represent MenH enzymes. The next closest homologue, an enol-lactonase from *Burkholderia xenovorans* has only 16% sequence identity with EcMenH and an rms difference of 1.96 Å over 217 common Cα-atom positions.

**Figure 7:**
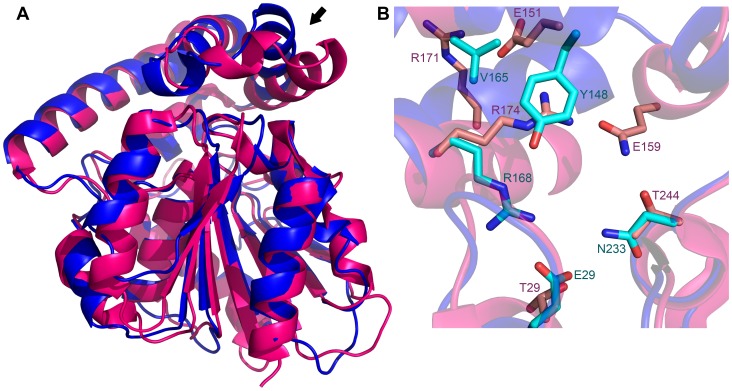
Structural variations between EcMenH and SaMenH. (A) Superposition of native EcMenH (blue) on to SaMenH (magenta). A region of significant divergence, the region from H6 to the end of H7, is indicated with an arrow. (B) Close up showing an overlay of the two active sites in the region from H6 to H7. The “conditionally conserved” residues discussed in the text are shown in stick representation, labeled with residue number and one letter amino acid code and colored in crimson for SaMenH and teal for EcMenH.

One significant difference between the EcMenH and SaMenH structures is in the positioning of a portion of the helical lid domain, from helix H6 through to the end of H7. This region is displaced down towards the loops of the core domain in SaMenH (Figure 7), and is associated with differences in the two active sites in the region around the chloride binding site of EcMenH (Figure 7B). In SaMenH Arg174, equivalent to Arg168 in EcMenH, is located where Tyr148 is in EcMenH. Tyr148 is replaced in SaMenH by Glu151, which has swung out of pocket to interact with Arg171 (equivalent to Val165 in EcMenH). These differences impact on the invariant active site tryptophan. In EcMenH, Trp147 sits between Trp131 and Tyr148, in an aromatic cluster, but the equivalent Trp150 in SaMenH is between Arg134 and Arg174. There is potential for flexibility in this part of the SaMenH active site, as Arg174 is hydrogen bonded only to water, whereas Arg168 in EcMenH is tightly constrained by its interactions with Tyr148 and Glu29.

**Figure 8:**
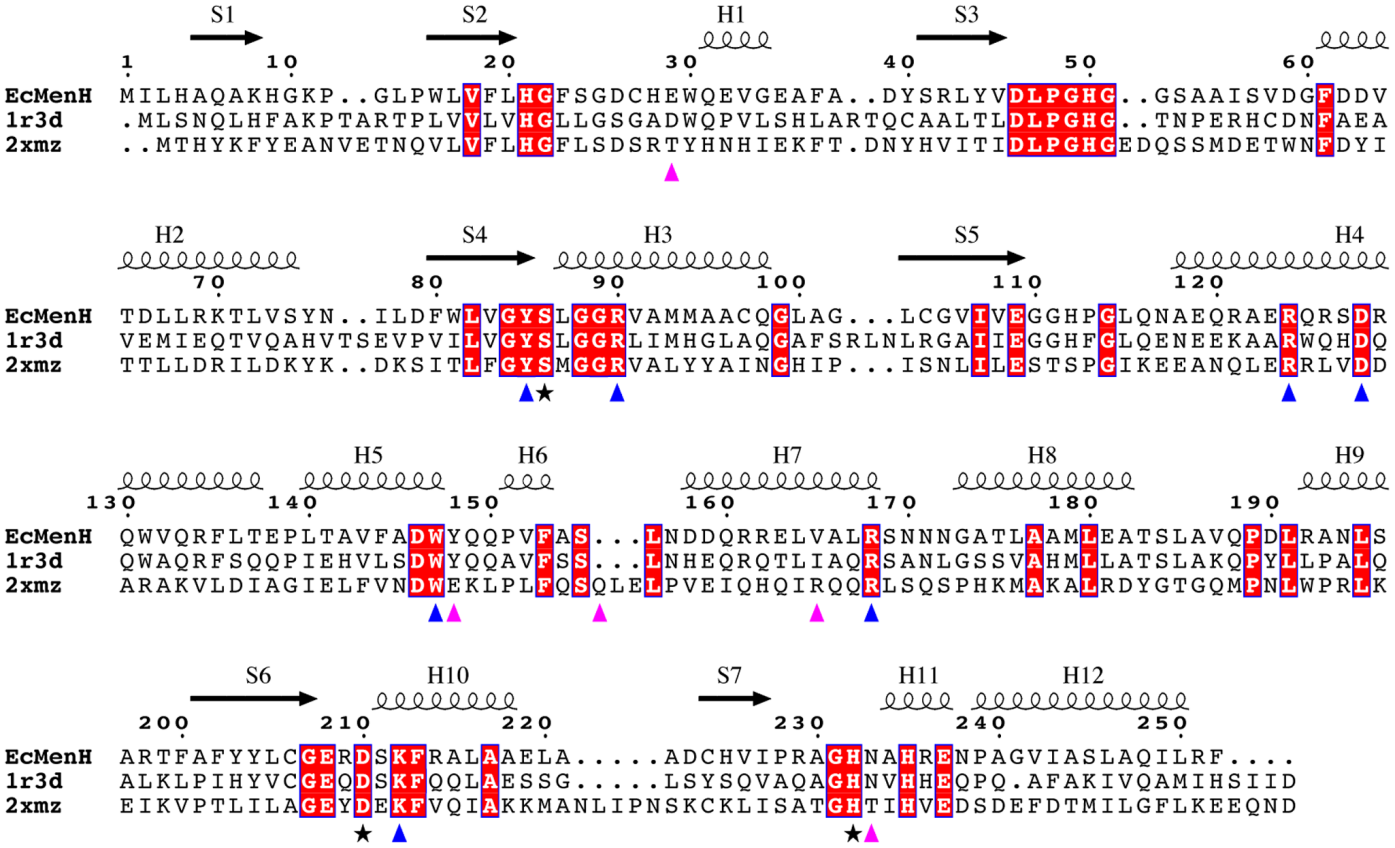
Sequence alignment of EcMenH, VcMenH (1r3d) and SaMenH (2zxm). Strictly conserved residues are boxed in red and catalytic residues are marked with a star. Other conserved residues identified as important in substrate binding or catalysis are marked with blue arrows and residues “conditionally” conserved and discussed above are marked with magenta arrows. The secondary structure for EcMenH is shown above the sequences. Alignment performed with ClustalW [38] and figure prepared using ESPript [39].

The structural variations in this part of the active site point to a pattern of conditional conservation in MenH enzymes of different species. We compared 35 MenH sequences in the NCBI conserved domain database MenH_SHCHC Tigr03695 family. This showed that the presence of Tyr at position 148 (EcMenH numbering) is correlated with a hydrophobic residue at position 165 (Figure 8). In contrast, substitution of Tyr148 by Glu or Gln as in SaMenH is always associated with an Arg at position 165 (171 in SaMenH). Presence of Tyr at position 148 is also consistently correlated with an Asp or Glu at position 29 and Asn at position 233. These residues, Tyr148, Glu29 and Asn233 mediate a network of interactions that fix Arg168 in place in EcMenH. In cases where Tyr148 is replaced by Glu/Gln, as in SaMenH, the equivalents of Glu29 and Asn233 are usually small residues (Ser/Thr/Ala). A 3-residue insertion in SaMenH further provides a Gln residue (Gln159) that interacts with the differently-positioned Arg174. This pattern of conditional substitutions is suggestive of perhaps two distinct conformations for this region in MenH enzymes that could be predictable by sequence alignment.

## Discussion

Enzymes belonging to the α/β-hydrolase superfamily pose a particular challenge for structure-function analysis. This fold supports a wide variety of catalytic functions, and high sequence diversity, making functional predictions difficult. Here we focus on the biosynthetic enzyme MenH, which has been biochemically characterized only in the case of the *E. coli* enzyme, EcMenH [9]. Sequence comparisons have identified other putative MenH enzymes, but also suggest very high sequence diversity. The crystal structure of EcMenH, presented here, provides a firm structural basis for interpreting the biochemical studies by Jiang *et al*. [9] on this enzyme. The high structural homology we demonstrate with the homologous enzymes from *V. cholerae* and *S. aureus* also helps to validate their annotation as MenH enzymes.

The natural substrate of MenH, SEPHCHC, has three carboxyl groups, contributed by its succinyl group, the central cyclohexenyl ring and the enol-pyruvyl group, which is eliminated in the enzymatic reaction. SEPHCHC is an unstable compound and all our attempts to prepare crystals of an EcMenH substrate complex, whether by soaking or by co-crystallization, were unsuccessful; the compound was found to have degraded. Our docking studies have, however, identified a general substrate binding mode that was particularly well defined when a transition state model was used. These docking studies also showed that the sites predicted for the cyclohexenyl and succinyl carboxyl groups coincide very closely with two anion binding sites that were occupied by sulfate and chloride ions in all the EcMenH crystal structures. These appear to be key anchor points for substrate binding to *E. coli* MenH. In this binding mode, which is consistent with manual docking of SEPHCHC to the *S. aureus* enzyme [12], the succinyl group is bound most deeply in the active site channel, the cyclohexenyl ring in the middle, adjacent to the catalytic Ser86, and the enol-pyruvyl group nearest the active site entrance, well situated for release following cleavage.

Two conserved basic residues, Arg90 and Arg124, are in position to bind the carboxyl groups of the enol-pyruvyl group and the cyclohexenyl ring, with Arg90 having a particularly important role. Its guanidinium group is fixed to the active site wall in a strategic position adjacent to Ser86. In the crystal structures it makes bidentate interactions with the sulfate ion, through NE and NH2, and in the docking studies it interacts similarly with the SEPHCHC cyclohexenyl carboxyl group. Arg124 projects out into the active site from the lid domain, held in position by a hydrogen bond with Asp128. Mutation of Arg124 to alanine has no effect on structure, as shown by our R124A crystal structure, and the significant changes in both *K*_M_ and *k_cat_* that accompany this mutation [9] must reflect its importance for productive substrate binding. In contrast, the conserved Lys212 has little impact on MenH kinetics [9] and from our docking studies does not seem to be significantly involved in substrate binding; its role may be to provide positive charge at the entry to the active site, for substrate capture. The most enigmatic of the conserved basic residues is Arg168. In EcMenH this residue forms part of the inner end of the active site pocket, where chloride binds, where it could potentially interact with the substrate succinyl carboxyl group. In our docking experiments, however, it is not close enough to interact with the substrate without adoption of an alternative conformation. Moreover the equivalent residue in SaMenH, Arg174, occupies a different position, packing against the conserved tryptophan (Trp147 in EcMenH) where Tyr148 does in EcMenH. This difference in location and structural roles between the two sequence-equivalent arginines in these structures brings into question the actual role this residue plays in the MenH mechanism.

One feature that emerges strongly from the present work is a preference in the docking studies for the succinyl carbonyl oxygen, which is negatively charged in the transition state of the reaction (Figure 2), to occupy the traditional oxyanion hole characteristic of α/β-hydrolase enzymes. This site, formed by the main chain NH groups of Phe23 and Leu87, is consistently occupied in our EcMenH structures by a well-ordered water molecule that makes highly favourable hydrogen bonds with the two NH groups. In contrast, in the proposed “unconventional oxyanion hole” the two hydrogen bonding groups involved, the indole NH of Trp147 and the phenolic OH of Tyr85, are consistently ∼7.5 Å apart in the EcMenH structures, too far to form an effective pair of hydrogen bonds with a substrate oxygen. The “traditional oxyanion hole” is proposed to be operative in the *S. aureus* enzyme [12] and seems likely to be a key feature of the catalytic mechanism.

What, then, are the roles of the conserved Tyr85 and Trp147 in MenH function? Mutations of these residues produce modest increases in *K*_M_ but large decreases in *k*_cat_ [9]. In our EcMenH structures, the indole NH of Trp147 and the OH of Tyr85 are ∼7.5 Å apart, well placed to bind the succinyl carboxylate between them, as shown in the docking studies. Mutation of either one of them may not compromise binding, especially as other residues such as Tyr148 and Arg168 could then become involved, but such mutations could compromise productive binding, thereby affecting catalysis. The loss of Trp147, which is at the centre of a large aromatic cluster, could severely affect the local structure in the active site, explaining the deleterious effect of the Trp147Ala substitution. Dawson *et al* suggested that the OH of Tyr85 may be involved in a C-H….O hydrogen bond, helping the catalytic histidine maintain the distribution of its positive charge, assisting its catalytic function [12] **.** This is consistent with the EcMenH structures, in which Tyr85 OH and His232 CD2 are between 3.1–3.4 Å apart, with the CD2 proton directed at the Tyr85 oxygen. If, as suggested [9], substrate binding is required before the catalytic triad can become active, Tyr85, which binds to both substrate and His232, could play a key role in activation of catalysis.

Finally, we have identified an intriguing pattern of sequence and structural variation at the inner end of the active site that suggests the existence of two sub-groups of enzymes within the wider MenH family. In one group, typified by EcMenH and the *V. cholerae* enzyme, a tyrosine at position 148, where it packs against Trp147 and interacts with the substrate succinyl carboxylate, is associated with a hydrophobic residue at position 165. Alternatively, a Glu/Gln at position 148 (EcMenH numbering), as in the *S. aureus* enzyme, is associated with an arginine at position 165 and other correlated changes. The invariant Arg168 (Arg174 in SaMenH) takes up quite different positions in the two sub-groups. These residues all impinge on the succinyl carboxylate binding site. They are associated with distinctly different local structures in the *E. coli* and *S. aureus* enzymes, suggesting the possibility of selective inhibition of MenH enzymes belonging to the two sub-groups.

### Conclusions

The structures of EcMenH and its two active site mutants Arg124Ala and Tyr85Phe reported here confirm that the enzyme adopts a typical α/β hydrolase core fold, with a helical lid. The structure is not perturbed by the mutations. The elongated active site is at the interface between the helical lid and loops of the core α/β domain. Two residues of the catalytic triad, Ser86 and His232 are located in the middle of this active site with other conserved residues, known to play a role in MenH binding or catalysis, on either side of them. A sulfate ion and a chloride ion are consistently bound in the active site, making interactions with a number of these key residues. Both the crystal structures and substrate docking studies point to the use of a conventional oxyanion hole, formed by the main chain NH groups of Phe23 and Leu87, rather than the proposed unconventional oxyanion hole involving Trp147 and Tyr85. Structural comparisons with SaMenH reveal differences in the active site around the chloride-binding site that may have their origins in a pattern of “conditional conservation” in two MenH sub-families.
